# Overcoming bioethical, legal, and hereditary barriers to mitochondrial replacement therapy in the USA

**DOI:** 10.1007/s10815-018-1370-7

**Published:** 2018-12-15

**Authors:** Marybeth Pompei, Francesco Pompei

**Affiliations:** 1 Northeastern University Bouvé College of Health Sciences, Boston, MA USA; 2 Exergen Corporation, Watertown, MA USA

**Keywords:** Mitochondrial replacement therapy, MRT, Three-parent babies, Reproductive autonomy, Mitochondrial Eve

## Abstract

The purpose of the paper is to explore novel means to overcome the controversial ban in the USA against mitochondrial replacement therapy, a form of IVF, with the added step of replacing a woman’s diseased mutated mitochondria with a donor’s healthy mitochondria to prevent debilitating and often fatal mitochondrial diseases. Long proven effective in non-human species, MRT recently performed in Mexico resulted in the birth of a healthy baby boy. We explore the ethics of the ban, the concerns over hereditability of mitochondrial disease and its mathematical basis, the overlooked role of Mitochondrial Eve, the financial burden of mitochondrial diseases for taxpayers, and a woman’s reproductive rights. We examine applicable court cases, particularly protection of autonomy within the reproductive rights assured by Roe v Wade. We examine the consequences of misinterpreting MRT as genetic engineering in the congressional funding prohibitions causing the MRT ban by the FDA. Allowing MRT to take place in the USA would ensure a high standard of reproductive medicine and safety for afflicted women wishing to have genetically related children, concurrently alleviating the significant financial burden of mitochondrial diseases on its taxpayers. Since MRT does not modify any genome, it falls outside the “heritable genetic modification” terminology of concern to Congress and the FDA. Correcting this terminology, the IOM’s conclusion that MRT is ethical, the continuing normalcy of the first MRT recipient, and increasing public awareness of the promising benefits might be all that is required to modify the FDA’s position on MRT.

## Introduction

Mitochondrial replacement therapy (MRT) is an innovative assisted reproductive method intended to prevent inherited mitochondrial disease, a maternally transferred heterogeneous group of diseases without curative treatment, and frequently fatal. Under the umbrella of MRT are four scientific methods: maternal spindle transfer (MST), maternal genome transfer (MGT), oocyte spindle transfer (OST), and pronuclear transfer (PNT), none of which can be legally undertaken in the USA. The purpose of this paper is to examine the reasons for the illegal status of MRT in the USA, and provide new evidence that supports elimination of the ban.

MST, MGT, and OST all involve removing the nucleus of a woman’s egg containing mutated mitochondria and transplanting the nucleus into the enucleated donor egg containing healthy mitochondria. The egg is then fertilized by the intended father’s sperm, and the resulting embryo is transplanted into the affected mother. OST was developed by a team at the New Hope Fertility Center in NYC and Guadalajara, Mexico, becoming the first of the methods to produce positive results, with a healthy baby boy delivered by Dr. John Zhang, the developer of the method.

Developed by a team from Newcastle University in the UK, PNT is an “embryo repair” technique in which two embryos are created via IVF. The first is created using an egg and sperm from the intended parents, and the second is created using a donor egg with healthy mitochondria and the father’s sperm. When the embryos are 1 day old, the pronuclei are removed from the first embryo. The majority of the mother’s diseased mitochondria is left behind in the enucleated embryo which is destroyed. Simultaneously, the pronuclei of the second embryo are removed and destroyed. The parents’ pronuclei are then placed into the second embryo, containing the donor’s healthy mitochondria. This constructed embryo would continue to develop, ultimately being transferred into the intended mother. Ethically and legally, PNT has been approved in the UK. However, the method is somewhat controversial due to the destruction of a human embryo.

PNT is legally provided for fertility treatment in Ukraine at the Nadiya Clinic. Four infants have been born through this method, the first in January 2017. According to the clinic’s director, Dr. Valery Zukin, four more infants have since been born, three of his patients are currently pregnant, and several others are going through the process. Dr. Zukin’s work was presented August 2, 2018, at the annual meeting of the European Society of Human Reproduction and Embryology held in Barcelona [1].

In February 2018, it was reported by the UK’s Human Fertilisation and Embryology Authority (HFEA) that two women carrying mutations in a gene that causes a rare condition known as myoclonic epilepsy with ragged red fibers (MERRF) [2] were approved to undergo PNT at the Newcastle facility. MERRF syndrome can be a devastating neurodegenerative disorder that worsens over time, often resulting in an early death. There is no cure.

In the USA, although MRT was determined ethically permissible in February 2016 following a 2-year investigation by the Institute of Medicine requested by the FDA [3], the method was summarily banned by the FDA. It is important to note that this ban was based on congressional funding restrictions on “heritable genetic modification” [4]. While deserving investigation, we believe Congress intended to refer to the commonly understood human attributes encoded by the nuclear DNA (nDNA) and not the mtDNA, which is not modified in MRT, and simply a transplant from a donor.

## Background

### First successful MRT procedure

MRT recently allowed a woman having suffered several miscarriages and the deaths of two children from Leigh syndrome, a deadly mitochondrial disease, to give birth to a healthy baby boy in April 2016. Given the ban against MRT in the USA, Dr. John Zhang, founder of New Hope Fertility Center in NYC, undertook the IVF part of procedure in NYC, and implantation part of the procedure in his clinic located in Guadalajara, Mexico, with IRB approval [5]. The FDA claimed that the IVF part of the procedure in NYC was a violation of the FDA’s ruling [6]. Instead of well-deserved congratulations, Dr. Zhang has been criticized by many of his peers, the media, and the FDA [7]. That this disease preventing achievement ever got to this point appears to be a consequence of a congressional ban with little or no basis in either science or ethics.

Fundamentally, MRT adds an additional step to in vitro fertilization (IVF), a clinically approved and successful procedure dating back to the 1970s, the first of which took place in Boston in 1944 [8]. Although no child was produced from these embryonic experiments, they led to 30 years of IVF development, culminating in the birth of the first “test tube” baby from IVF, Louise Brown, who was born in the UK in 1978. Since that time, more than 8 million babies have been born worldwide from IVF [9]. In the USA, as of 2014, the latest data available, nearly 1 million babies were born via IVF [10].

In MRT, a technique based on IVF, the nucleus from an afflicted mother’s oocyte is implanted into an enucleated oocyte from a healthy donor, and fertilized by the intended father. The resulting embryo is then transplanted into the afflicted mother. Although termed “three-parent baby” by the press, this is incorrect and misleading. The “third parent,” the donor female, contributes only mtDNA, the cell’s microscopic power plants. While generating more than 90% of the energy required by the body, mtDNA contributes none of the traits to the child based on personal or socially valued characteristics, all of which are resident in the nDNA [11]. These diminutive mtDNA power plants are, however, subject to more than 400 known pathogenic mutations causing severe and often fatal diseases [12]. As considerable energy is required by the brain, heart, lungs, and muscles, these are the first areas affected by mitochondrial diseases. MRT research on animal and donated human embryos suggest pathogenic mtDNA carryover from technically undetectable levels to less than 2% [13–16]. Given that mitochondrial diseases manifest clinically only when the mutated mtDNA threshold is 60% or higher, levels lower than 2% are highly unlikely to cause disease in children [17, 18].

### The scourge of mitochondrial disease

Mitochondrial disease can affect almost any part of the body including the blood cells, brain, ears, eyes, heart, kidneys, liver, muscles, nerves, and pancreas. Currently, there are 41 known diseases directly linked to mitochondrial dysfunction, few of which would be recognized by many, none of which are curable [19]. Further, mitochondrial disease has recently been connected to a wide range of well recognized metabolic and degenerative diseases including Alzheimer’s, ALS, blindness, cancer, deafness, diabetes, epilepsy, Huntington’s disease, mental retardation, obesity, and Parkinson’s disease [20–26]. Symptoms typically begin to appear in the toddler and preschool years, but adult onset is becoming increasingly common. Prognoses range from progressive weakness to death. There are no cures.

Mitochondrial disease is complicated and difficult to diagnose since it does not always manifest in people who have the mtDNA mutations. Both sexes can harbor the mutations or have a mitochondrial disease, yet a male does not transmit his mtDNA to his children, it is solely transmitted maternally. Given the devastating and deadly results of this disease, MRT, if proven effective, represents an extraordinary opportunity for women who are carriers to have their own genetic children and restart their genetic line on a healthy basis. More than 1 in 200 healthy humans harbors a pathogenic mtDNA mutation that can result in a mitochondrial disease for the carrier, and if female, the mutations would be inherited by her children [27]. According to Boston Children’s Hospital, mitochondrial disorders affect between 1 in 6000 and 1 in 8000 live births, making mitochondrial disease almost as common as childhood cancer [28]. According to the CDC, 10.3% of people of all ages in the USA are in fair to poor health [29]. Using the 2012 US census for convenience, the population was 314 million people, 282 million of which were healthy. Applying the 1 in 200 estimate referenced above would bring the potential number of individuals unknowingly harboring a pathogenic mtDNA mutation to 1.4 million, 700,000 of whom are female, approximately one third of whom are of reproductive age. It is important to note that mitochondrial disease affects multiple organs, multiple family members, and multiple generations.

### Human clinical trials

Over the past several years, many animal trials of MRT have been undertaken ranging from mice to primates to assess safety and ethical questions supporting the many pending requests to initiate human clinical trials in the USA [30, 31]. Most recently, the success of primate studies provides strong evidence supporting the effectiveness of MRT in eliminating mitochondrial disease in humans [32–34]. Further evidence demonstrates continuing with animal trials, as suggested by the FDA, may not be ethically justifiable. Models are imperfect, and cell-based studies have evidenced only limited clues about possible long-term side effects applicable to humans [35–38].

### Updating a successful outcome

Seven months following the birth as a result of MRT, Dr. Zhang reported the baby was healthy and asymptomatic. The mother, a Jordanian, carries the mitochondrial mutation for Leigh disease, a deadly progressive neurological disease affecting the central nervous system [39]. Prior to the MRT procedure, she had four miscarriages and suffered the loss of two children to Leigh disease. Although not known whether applicable in this case, consanguineous marriage is part of the cultural and social life for most Arab countries. First cousin marriages are preferred [40], significantly increasing the risk of their progeny inheriting lethal genetic conditions. Of interest, consanguinity has recently been associated with autosomal recessive diseases such as Leigh disease, and more recently with recurrent miscarriages [41–43]. On April 22, 2017, Dr. Zhang requested a meeting with the FDA for an investigational new drug application (IND) for “spindle transfer for assisted pregnancy in patients with mitochondrial disease.” This was denied. On August 4, 2017, the FDA informed Dr. Zhang that he was in violation both for creating the embryo as well as exporting the embryo to Mexico for implantation, demanding a written response addressing their claims of violation and how he will prevent any recurrence [6]. Not surprisingly, these reports are raising concern that the congressional ban will force more researchers from the USA to practice their skills in other countries. Commenting on the courage it took for the scientists and physicians to undertake these treatments in Mexico, Dr. Dieter Egli, a researcher skilled in the art, is quoted as saying “I think there is an imbalance in regulation and oversight in some places, putting novel treatments on the long bench, and therefore it had to be done that way” [44].

## Ban on heritable genetic modification

### First-in-human clinical investigations deemed ethical—yet banned by the FDA

As previously stated, on February 3, 2016, following a 2-year study of the safety and ethics of MRT, the IOM, now known as the National Academy of Medicine (NAM), rendered a comprehensive albeit guarded approval for MRT that would permit human research on MRT to ethically move forward in the USA. The IOM wrote: “The replacement of whole, intact, and naturally occurring mitochondrial genomes represents a qualitatively different form of heritable genetic change from that resulting from any approach for modifying nDNA, which would likely involve editing rather than en bloc replacement of chromosomes-the closest parallel to MRT” [45]. Yet, interestingly, the IOM termed the procedure a “heritable genetic modification.” Immediately following the announcement, the FDA Advisory Committee, citing current budgetary law limitations, ruled that such research could not be performed in the USA. Yet, the FDA actually received a significant budget approval from Congress for FY2016 representing a 5% budgetary increase, but, with a ban on “heritable genetic modification.” In announcing the ban, the FDA stated: “Such research ... is still years from producing therapies that regulators would have to green light before they could be tested in humans” [46].

The report from the IOM evidences a significant oversight in using the term “heritable genetic modification” to describe MRT. While both the nDNA from the mother and mtDNA from the donor are heritable in MRT, there is no heritable “genetic modification” in MRT—neither the mother’s nDNA, nor the donor’s mtDNA is modified in any way. The procedure involves a transplant of donated intact unmodified mitochondria in a procedure paralleling egg donation, providing the intended mother the means to have a genetically related child. As such, it appears the IOM used inappropriate terminology which was incorporated into the Consolidated Appropriations Act, which led to banning MRT in the USA [47]. This needs to be challenged.

### MRT in the UK

In February 2015, following approximately 10 years of research, considerable societal, religious, and political interaction, and the overwhelming approval of both houses of Parliament, the UK became the first country in the world to approve laws allowing MRT. These took effect on October 29, 2015. Recognizing the technical and ethical complexities of MRT, the Human Fertilisation and Embryology Authority (HFEA), the government entity controlling licensing and monitoring of all UK fertility clinics and embryonic research, was charged with ensuring a robust licensing process. The Nuffield Council on Bioethics, an independent body advising policy makers, determined it morally acceptable for women at risk of passing on mitochondrial disorders to be offered MRT, provided techniques are proven sufficiently safe and effective, and that an appropriate level of information and support is offered [48].

### Different terminology for different procedures

Terminology should be procedure specific, yet there appears to be a lack of consensus in the terminology describing genetic procedures. To clarify, genetic modification occurs when the sequence of the genome is altered (gene editing/engineering) using a system such as CRISPR/Cas9 genome editing [49]. Ethically, this would be a valid source of concern. However, MRT does not involve genome modifications, nor does it provide a path for doing so [17]. Accordingly, using the term genetic engineering to describe MRT would be inaccurate and misleading. MRT is a genetic recombination, which is actually no different from what occurs naturally in sexual reproduction when germ cells recombine different alleles both before and during fertilization. This is a pivotal distinction [50]. The UK experience is instructive on the question of the term “genetic modification.” The Department of Health determined that MRT does not involve genetic modifications, arguing as we do, that “The techniques are viewed as replacing faulty mitochondrial genes, while leaving both the nuclear DNA and mitochondrial DNA intact,” but leaves the possibility of revisiting this issue [51]. As the UK legalized MRT after 10 years of study, there is a presumption of broad public support for this decision.

Unfortunately, while holding the promise of a significant scientific breakthrough, MRT is incorrectly considered by many as a heritable genetic modification in which changes would be passed on to succeeding generations in perpetuity. This perception raises social, ethical, and legal concerns, particularly given the long-standing international consensus against heritable genetic modification. We believe this perception is scientifically incorrect as discussed below. The heritable factor is self-limiting, ceasing with the last female born in an unbroken lineage of at least one female per generation to an affected mother employing MRT.

### Previous views of MRT lacking genetic risks

Perhaps it is the term “DNA” in mtDNA that causes MRT to be incorrectly placed in categories such as genetic engineering or gene therapy, anxiety-causing terms for many. Differentiating: MRT involves an actual transplant of a functional organ at the cellular level; genetic engineering is a targeted method of introducing desirable traits into an organism by altering the genome; gene therapy describes the attempt to cure or prevent disease by altering or replacing defective genes in an otherwise healthy genome. MRT does not alter or replace genes in a genome, rather the entire mitochondrial genome is replaced, analogous to a solid organ or bone marrow transplantation. MRT preserves the natural state of both the mitochondrial and nuclear genomes intact, thus eliminating inherent ethical risks in opening and altering a genome.

Stressing that MRT was quite separate from the illegal act of altering DNA in the nucleus, Dame Sally Davies, Chief Medical Officer for England and Chief Medical Advisor to the UK, provided an elegant explanation of the method to Parliament: “This is about changing the battery pack, it’s not about touching the chromosomes that make us what we are. The nucleus is sacrosanct and it is illegal to touch it” [52].

MRT prevailed in the UK mainly due to the involvement of the stakeholders: its government, citizens, clergy, medical practitioners, and, most importantly, women afflicted with diseased mitochondria [53]. In the USA, possibly due to the headline-grabbing connotation of “three-person parents” by the press, many individuals mistakenly consider MRT to be immoral and unethical. “But not so,” claimed Dr. Arthur Caplan, a well-respected medical ethicist. In a televised interview a few years ago referring to MRT, Dr. Caplan said the technique is “not without its risks, but it’s treating a disease” and that preventing a disease that can be passed down for generations would be ethical “as long as it proves to be safe” [54].

## Reproductive liberty, legal and social acceptance

### A judicial precedent

The IOM recommended initially limiting MRT to male only embryos to eliminate the heritability factor. However, it is not simply a matter of heritability at issue. Inasmuch as both sexes can harbor the mutations, or actually have the disease, excluding female embryos would “reinforce discriminatory and sexist stereotypes towards women by devaluing females” according to the WHO [55], and is illegal gender selection in a number of countries [56]. The WHO also states that reproductive health rights of couples “… includes their right to make decisions concerning reproduction free of discrimination...” [57].

The panel raised concern that the yet-to-be-born individuals would have no decisional capacity to choose or refuse MRT. However, as none of us walking the earth today had a say in selecting either parent prior to our birth, we believe this concern is without standing. The IOM also recommended limiting inclusion in the initial clinical trial to women known to carry pathogenic mtDNA. Interestingly, a judicial precedent from Roe v. Wade may be applicable. Legally and ethically, it should be the reproductive right of any woman concerned she may carry the mutation to proceed with MRT.

Certain commonly accepted genetic tests such as preimplantation genetic diagnosis (PGD) have gender selection implications, such as with X-linked genetic diseases, and therefore possibly subject to the ethics of discriminatory and sexist stereotypes to women. However, these diagnoses are for embryos with indications of the actual disease, not a rejection of all embryos of a specific gender, and therefore not an ethical issue by rejecting all females. For X-linked genetic diseases, females are the affected gender, but not all female embryos are rejected—only those with the disease. The IOM recommendation rejects MRT for all females because all females might pass pathological mtDNA on to future generations, which is very different ethically.

Women known to carry pathogenic mtDNA may already have standing to challenge the FDA’s ban on MRT based on the FDA’s own regulations on expanded access (sometimes called “compassionate use”) [58] as follows:

“Expanded access (sometimes called “compassionate use”) is the use of investigational drugs, biologics or medical devices outside the clinical trial setting for treatment purposes. Expanded access may be appropriate when all the following apply:

- Patient has a serious disease or condition, or whose life is immediately threatened by their disease or condition.
- There is no comparable or satisfactory alternative therapy to diagnose, monitor, or treat the disease or condition.
- Patient enrollment in a clinical trial is not possible.
- Potential patient benefit justifies the potential risks of treatment.
- Providing the investigational medical product will not interfere with investigational trials that could support a medical product’s development or marketing approval for the treatment indication.”

More recently, the “right-to-try law” of May 2018 [59] streamlines the process by eliminating some of the FDA steps with respect to drugs, but firmly establishes the political support for the ethics of permitting patients to try therapies not yet approved by the FDA. Importantly, the FDA’s own expanded access program is not limited to drugs, which appears to open the door to MRT. Any child of women with diseased mitochondria has a high probability of incurring incurable mitochondrial disease, which is a terminal illness. Accordingly, given the constitutional support for reproductive freedom, congressional support for right to try, and the FDA’s own “compassionate use” history, it may be a small step for the FDA to allow patients and their physicians to bypass the FDA’s ban on MRT and support the idea of extending expanded access to include the unborn at serious risk.

In general, excepting the eugenics era in the USA in the late nineteenth and early twentieth century when many citizens were forcibly subjected to compulsory sterilization, the USA has not interfered in the reproduction rights of its citizens, even to the point of legalizing abortion. Until now that is. While not rising to the level of injustice as compulsory sterilization, in denying women and men the opportunity to have healthy genetically related children, the government’s ban on MRT has a similar impact by violating a parental right to genetic affinity, a potential legal right recently upheld by Singapore’s Supreme Court in which the court created a completely new category of loss—“genetic affinity” [60].

### A woman’s constitutional right to reproductive autonomy and privacy

Arguably, the government’s ban on MRT is in direct violation of a woman’s constitutionally protected rights to privacy and reproductive autonomy, encompassing both positive and negative rights. The positive right being her freedom to decide whether or not to have children, while the negative right is her freedom to prevent the conception or birth of afflicted children. Legally, these rights have been upheld by the Constitution of the USA, Roe v. Wade, Planned Parenthood v. Casey, and others. While not specifically cited in the Constitution, in Roe v. Wade, Section VIII [61], the Supreme Court of the USA (SCOTUS) recognized that a right of personal privacy, or a guarantee of certain areas or zones of privacy, has existed under the Constitution since 1891 [62]. Eighty years later, in 1972, SCOTUS firmly established the right to privacy as being fundamental and that any governmental infringement of that right must be justified by a compelling state interest. In support, SCOTUS referenced the roots of that right in the First Amendment [63], the Fourth and Fifth Amendments [64] in the penumbras of the Bill of Rights and the Ninth Amendment [65], and in the concept of liberty guaranteed by the first section of the Fourteenth Amendment [66].

Under Roe v. Wade, women have the legal right to abort their unborn and unwanted children, a right protected and supported by the government. In direct contrast, women are prohibited by the government’s mandate from assuring the health and wellbeing of their unborn and wanted children through MRT, actions that are logically incongruous. Arguably, the findings in Roe v. Wade protecting a woman’s right to privacy and her zone of privacy would, at the same time, protect a woman’s right to choose MRT.

### An ethically acceptable solution?

According to the IOM’s report, were MRT male specific, a viable solution would possibly be at hand, passing ethical, legal, and social scrutiny. Although pathogenic mtDNA is inherited maternally by both male and female children, it is only the female children who pass the mutated DNA along to their children. Still, while the male may not pass pathogenic mtDNA mutations to his children, both males and females are subject to any of the long list of diseases now associated with mutated mtDNA. As such, MRT would be equally beneficial to both sexes, rendering a male only concept prejudicial and maleficent. Moreover, postulating the destruction of female embryos involved in gender-specific selection raises further ethical challenges such as gender bias and establishing embryonic personhood.

### Recent societal acceptance

Since congressional funding and congressional lifting of the ban on MRT is necessary for the FDA to advance MRT, public opinion is a necessary part of the success of MRT. In March 2016, the first of its kind survey in the USA interviewed women directly affected by pathogenic mtDNA and those at risk of carrying pathogenic mtDNA mutations, measuring the level of support for oocyte mitochondrial replacement therapy (OMRT) to prevent transmission of mutated mitochondria [67]. All of these women were aware of the risks involved, with 78% considering the risks high enough for them to consider not having children at all. Ninety five percent of these women believed development of MRT to be important and worthwhile. Of the prospective oocyte donors also surveyed in the same study, 87% indicated they would provide healthy eggs. In April 2016, a compilation of surveys over the past 30 years demonstrated 65–87% of Americans approve of “gene therapy” for curing a fatal or usually fatal disease; 77–78% approve of reducing the risk of a fatal disease developing later in life; 64% believe the federal government should fund research for developing “gene therapy” treatments; 59% believe the FDA should approve gene therapy [68].

### Economic concern

Economic concern has been raised for the costs involved to develop and support MRT, assuming it would be applicable for relatively few women. This concern appears shortsighted. Given the growing list of diseases linked to pathogenic mitochondria affecting both males and females, and the taxpayer borne costs for care and support of those afflicted, the benefits would significantly outweigh the costs involved [53]. Further, if mitochondrial related diseases can be mitigated through MRT, the financial burden to taxpayers would be mitigated in tandem. Critics are mainly concerned altering DNA genomes might lead to unintended consequences, possibly detrimental to future progeny. The fact that the UK has thoroughly considered this issue for a number of years and concluded that the risks were manageable, suggests that the critics perhaps should reconsider their concerns.

### The importance of genetically related children

For many women, the importance of carrying and delivering genetically related children compared to adoption or donor egg IVF cannot be overstated. Many women would, and have, put themselves in harm’s way in order to do so, thus raising the question of ethical justifiability. For example, consider the ten young women and donors who put themselves at significant risk [69] by enrolling in a study of uterine transplantation at the Cleveland Clinic, a lengthy surgical process. The first young woman underwent a 9-h surgery in February 2016. The procedure ended in failure a few weeks later due to infection, requiring additional surgery to remove the implanted uterus [70].

Notwithstanding failure, the first baby born in the USA as a result of a transplanted uterus, which was donated by a nurse, was reported to have occurred at Baylor University Medical Center in Texas in 2017 [71]. As a live organ transplant, immunosuppressant medication during the pregnancy was mandatory. To eliminate continuation of immunosuppressants, the transplanted uterus requires removal following the delivery. While controversial from ethical and economic perspectives given the risks to both women and the costs involved, eight other women have recently received a donated uterus at Baylor, clearly demonstrating the fundamental importance of carrying and delivering a genetically related child for the mother. As reported by Robertson, “In most places the choice is not between uterus transplant and gestational surrogacy, but uterus transplant or no genetic child at all” [72].

## Heritability

The remaining issue of concern, a recurring subject in the FDA meetings and woven throughout the IOM report, is the fear of heritability of unknown genetic effects on future progeny. Unquestionably, new genetic technologies are often met with skepticism and fear regarding the hereditability factor. Much of this fear can likely be traced to the increasing knowledge of heritable genetic diseases. The NIH and other medical institutions list hundreds of known and very serious heritable genetic diseases, offering panels of tests to search for these genetic markers of disease [73, 74]. As such, societal fear of disease heritability caused by genetic manipulation is understandable. It is incumbent upon supporters of MRT to draw a clear distinction between MRT, a cell organ transplant with limited inheritance, and genetic alteration of nDNA which can profoundly affect future generations.

### Alleviating fear of the unknown microchimerism

Interestingly, there may be another beneficial similarity between body organ and cell organ transplants that could alleviate fear of the unknown heredity factor: microchimerism. Microchimerism, the co-existence of donor and recipient DNA in hematopoietic elements, has been a subject of interest for researchers dating back to the 1950s, with observed benefits rising in promoting tolerance in organ transplantation [75], and in long-term survival patients with Fanconi anemia [76], a rare genetic disease associated with mitochondrial dysfunction. This phenomenon can occur following blood transfusions, organ transplants, or pregnancies, and could have an influence on tolerance, prolonged organ survival, or the rejection process [77]. Further, donor DNA has long been detected in recipient blood following bone marrow transplantation [78, 79] and in recipients for years following kidney transplantation [80], as well as in upper extremity transplantation for our wounded warriors [81]. With greater acceptance of the idea of commonly existing microchimerism, the fear of a different person’s mtDNA as a small fraction of the total DNA in a person becomes more acceptable.

### Limited inheritance: the role of Mitochondrial Eve and her daughters

Given the issue of concern over heritability, it is important to consider the actual science of mtDNA inheritance in the female line. In a 1987 worldwide sample of 147 people, scientists concluded that all mtDNA appears to stem from one woman who likely lived in Africa about 200,000 years ago [82]. She was immediately labeled “Mitochondrial Eve” by the media, a catchy term still used today, although mistakenly conflated by some with “Biblical Eve.” Yet unlike Biblical Eve, Mitochondrial Eve had a mother, and so on before her. Further distinguishing Mitochondrial Eve is that she is the most recent common ancestor of all humans through the female line, evidenced by certain common characteristics of the mtDNA of all humans.

Although many have examined the time and location of Mitochondrial Eve, the basic idea of her existence is well accepted, and evidences only one inherited line of mtDNA which is specific to all females today [83–85]. This simple statement has two important consequences: (1) all other female lines of mtDNA became extinct at some point in the past, demonstrating transplanted mtDNA by MRT in female offspring is not necessarily passed on to future generations forever and is overwhelmingly likely to end in a finite number of generations. (2) All females are related to each other by their mtDNA. Unlike mtDNA, female nDNA is demonstrably different due to the contribution of male nDNA each generation, thus normal familial relatedness is very remote. However, inasmuch as all females are mitochondrial daughters of Mitochondrial Eve, they may be considered mitochondrial sisters in regard to mtDNA donation. Accordingly, transplanting mtDNA from an egg donor to an afflicted mother’s egg may have considerably less risk than previously assumed. As far as we can tell, this potentially important aspect has not been studied.

A recent study conducted on humans and a variety of animals including mice and macaques examined the potential problem of placing mtDNA into a novel genetic background causing harmful effects due to deleterious interactions between the mitochondrial and nuclear genomes. The authors concluded that even with cross-strain mtDNA to nDNA environments, there was “little tendency for introgression of mtDNA to be harmful” [86]. Corroborating findings examining human nuclear-mitochondrial mismatches also concluded mismatches are unlikely to jeopardize the safety of MRT [87].

### Finite number of generations for MRT

Addressing mtDNA extinction, item 1 above, a mtDNA transplant to a female might be considered the initiation of a new mtDNA line, yet begs the question as to how many generations it is likely to last. This mathematical problem was addressed 140 years ago by Galton and Watson when they considered the identical mathematical problem of the extinction of family names inherited only through male lineage [88]. Their solution, the Galton-Watson process, is an important mathematical method of addressing similar population issues [89]. The most important parameter in the calculation of the extinction probability of a male family name is the average number of sons had by each man. The Galton-Watson process demonstrates that if the number of sons per man averages > 1, then it is possible for the family name to last forever. This does not mean certainty, only that it is possible. Conversely, if the average number of sons per man is ≤ 1, the family name, with certainty, will then become extinct. Similarly, if the average number of daughters per woman in the new MRT mtDNA line is ≤ 1, it will become extinct in a finite number of generations. Given the continuous decline in the average number of children per woman in today’s culture, the extinction of a new MRT mtDNA line might be quite short, on the order of a few generations, thus limiting the effect of heritability.

**Fig. 1:**
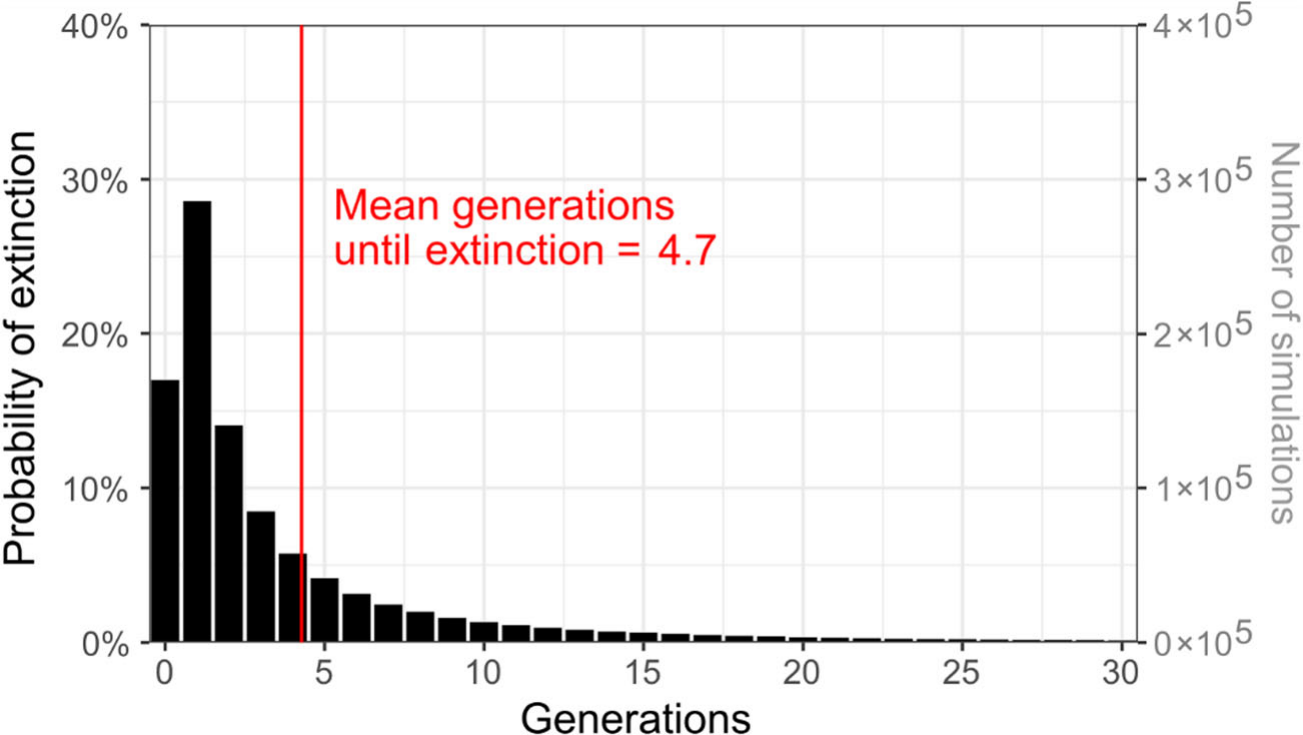
Number of generations until extinction of a new mtDNA line transplanted into a single female recipient: simulated results

To calculate the number of generations to extinction, it is necessary to use the full distribution of number of offspring per woman, as obtained from the US Census Bureau [90] and the CDC [91]. As a consequence, the methods become more complicated than applying a specific formula. We obtained census and birth certificate data on the number of offspring that women living in the USA had, as of 2016.^1^ The average number of daughters per woman was 0.95. Applying the Galton-Watson process to the data,^2^ the number of generations until extinction of a female-transplanted mtDNA line was 4.7 on average, and 90% of cases were under 12 generations (Fig. 1). It should be noted that these calculations are approximations, since the Galton-Watson process relies on several simplifying assumptions.

## Conclusion

Allowing MRT in the USA would save lives, avoiding costly expenditures, and provide the women likely to transmit a deadly mitochondrial disease, a method by which they can have a healthy child. Hopefully, many other physicians will be able to follow in the footsteps of Dr. Zhang in providing this life-saving therapy to the many women who would benefit. The ethical, legal, social, and financial ramifications surrounding the congressional prohibition of MRT in the USA have been examined in depth. The previously overlooked evidence supporting the benefits of MRT, applicable to yet unknown millions of individuals, arguably, outweighs the prohibition. Since MRT does not modify any genome, it arguably falls outside the “heritable genetic modification” terminology of concern to Congress and the FDA. A correction of this terminology, the IOM’s conclusion that MRT is ethical, the continuing normalcy of the first MRT recipient, and increasing public awareness of the promising benefits might be all that is required to modify the FDA’s position on MRT to be similar to the regulations applied in the UK. We heavily weighted the fact the UK studied the above issues for 10 years, with much broader input from stakeholders as well as scientists; since in the end, this new therapy is an evaluation of risk to the stakeholders. The UK concluded that the risk of heritable genetic modification via MRT was acceptably small, especially compared to potential benefits. We further reduce the risks associated with MRT from the UK acceptance level, to a level significantly smaller due to the limited heritability demonstrated above. Now, with MRT becoming available in Mexico, UK, Ukraine, and other countries, will US women with mutated mitochondria claiming their reproductive rights to healthy genetically related children be forced into a new form of “transplant tourism”?

## Footnotes

1 To estimate the distribution of total offspring per woman, we used data from women at early post-reproductive ages. For US women age 45–50, year 2016 data are available on offspring per woman [85] and the overall offspring sex ratio [86]. Sex-specific offspring distributions were obtained from these data by assuming sex ratio is independent of offspring numbers, which likely holds approximately but not exactly.

2 To investigate the number of generations from mtDNA transplantation to a female recipient until extinction of the new mtDNA line, we simulated one million runs of the Galton-Watson process with offspring distributions taken from the year 2016 US data. Each run of the simulation was adjusted for the possibility that there could be a final, male only generation of carriers of the new mtDNA line.
